# Immune responses to SARS-CoV-2 infection in hospitalized pediatric and adult patients

**DOI:** 10.1126/scitranslmed.abd5487

**Published:** 2020-10-07

**Authors:** Carl A. Pierce, Paula Preston-Hurlburt, Yile Dai, Clare Burn Aschner, Natalia Cheshenko, Benjamin Galen, Scott J. Garforth, Natalia G. Herrera, Rohit K. Jangra, Nicholas C. Morano, Erika Orner, Sharlene Sy, Kartik Chandran, James Dziura, Steven C. Almo, Aaron Ring, Marla J. Keller, Kevan C. Herold, Betsy C. Herold

**Affiliations:** 1 Department of Microbiology and Immunology, Albert Einstein College of Medicine, Bronx, NY 10461, USA.; 2 Department of Immunobiology, Yale University, New Haven, CT 06520, USA.; 3 Department of Pediatrics, The Children’s Hospital at Montefiore and Albert Einstein College of Medicine, Bronx, NY 10461, USA.; 4 Department of Medicine, Montefiore Medical Center, Bronx, NY 10467, USA.; 5 Department of Biochemistry, Albert Einstein College of Medicine, Bronx, NY 10461, USA.; 6 Department of Pathology, Montefiore Medical Center, Bronx, NY 10467, USA.; 7 Department of Emergency Medicine and Biostatistics, Yale University, New Haven, CT 06520, USA.; 8 Department of Internal Medicine, Yale University, New Haven, CT 06520, USA.

## Abstract

Children and youth infected with severe acute respiratory syndrome coronavirus 2 (SARS-CoV-2) have milder disease than do adults, and even among those with the recently described multisystem inflammatory syndrome, mortality is rare. The reasons for the differences in clinical manifestations are unknown but suggest that age-dependent factors may modulate the antiviral immune response. We compared cytokine, humoral, and cellular immune responses in pediatric (children and youth, age <24 years) (*n* = 65) and adult (*n* = 60) patients with coronavirus disease 2019 (COVID-19) at a metropolitan hospital system in New York City. The pediatric patients had a shorter length of stay, decreased requirement for mechanical ventilation, and lower mortality compared to adults. The serum concentrations of interleukin-17A (IL-17A) and interferon-γ (IFN-γ), but not tumor necrosis factor–α (TNF-α) or IL-6, were inversely related to age. Adults mounted a more robust T cell response to the viral spike protein compared to pediatric patients as evidenced by increased expression of CD25^+^ on CD4^+^ T cells and the frequency of IFN-γ^+^ CD4^+^ T cells. Moreover, serum neutralizing antibody titers and antibody-dependent cellular phagocytosis were higher in adults compared to pediatric patients with COVID-19. The neutralizing antibody titer correlated positively with age and negatively with IL-17A and IFN-γ serum concentrations. There were no differences in anti-spike protein antibody titers to other human coronaviruses. Together, these findings demonstrate that the poor outcome in hospitalized adults with COVID-19 compared to children may not be attributable to a failure to generate adaptive immune responses.

## Elucidating immune responses in COVID-19

Compared to adults, young people with COVID-19 have milder disease. Pierce *et al.* compared immune responses in hospitalized adult and young patients with COVID-19 to identify potential contributing mechanisms. In the first week after hospitalization, circulating IL-17A and IFN-γ concentrations were inversely related to age. More than 3 weeks later, CD4^+^ T cell responses to viral spike protein were higher in adult compared to younger patients. Neutralizing antibody titers were also higher in adults and correlated positively with age and negatively with IL-17A and IFN-γ. These findings suggest that the poor outcome in adults is not caused by a failure to generate adaptive immune responses.

## Teaser

Differences in immune responses to SARS-CoV-2 in children and adults are linked to clinical outcomes.

## INTRODUCTION

The World Health Organization (WHO) declared coronavirus (CoV) disease 2019 (COVID-19) a pandemic in March 2020, and as of 15 September 2020, there have been more than 29 million cases worldwide with more than 929,000 deaths (*1*). The reasons for this rapid spread include the ability of the virus to transmit from human to human and an immunologically naïve population. Many infected individuals have poor outcomes, and the need for hospitalization continues to overwhelm the medical system with mortality rates exceeding 8% at many sites (*2*, *3*). New York City was an early epicenter of the COVID-19 infection in the United States, and in the Bronx, there have been more than 52,000 cases and 3992 deaths as of 13 September 2020 (*4*).

The causative agent of COVID-19, severe acute respiratory syndrome coronavirus 2 (SARS-CoV-2), is the newest member of the *Betacoronavirus* family, which also includes the causative agents of SARS-CoV-1 and Middle East respiratory syndrome virus (MERS) (*5*). These viruses are genetically distinct from the common cold human CoVs (HCoV) such as 229E and OC43, which infect children and adults with little or no morbidity. All of the CoVs originated in animals, but SARS-CoV-2 presumably crossed the species barrier quite recently and is distinct from both SARS-CoV-1 and MERS because it has adapted to easily spread from human to human.

One distinguishing feature of the newer CoVs (SARS-CoV-1, SARS-CoV-2, and MERS) is that regardless of geographical location, children and youth have milder disease and progress to acute respiratory distress syndrome (ARDS), a hallmark of morbidity with COVID-19, less often than do adults (*6*, *7*). For example, in a study of 2143 pediatric patients in China with confirmed (*n* = 731) or suspected (*n* = 1412) COVID-19, over half had mild disease and <1% had severe or critical disease (*8*). The markedly reduced incidence of severe respiratory disease in children with COVID-19 contrasts sharply with other viruses such as respiratory syncytial virus where the burden in young children is much greater (*9*). The differences in clinical outcomes between children and adults with COVID-19 suggest that age-dependent host features are important contributors to the pathophysiology of the disease.

An exception to mild manifestations of COVID-19 in pediatric patients is the newly emerging, multisystem inflammatory syndrome in children (MIS-C). This syndrome occurs in a minority of patients <21 years of age who are infected with COVID-19 and is characterized by fever; clinical laboratory evidence of inflammation; serologic evidence of recent SARS-CoV-2 infection; and any combination of cardiac, renal, respiratory, hematologic, gastrointestinal, dermatologic, or neurological disease (*10*, *11*).

The basis for the overall relatively mild clinical manifestations of COVID-19 in children compared to adults and the immunologic features that distinguish children with and without MIS-C are unknown. However, data from an earlier SARS outbreak with the genetically related virus, SARS-CoV-1, may provide insights. Patients who died from SARS-CoV-1, which was self-limited because the virus did not adapt as well to person-person spread, had high serum concentrations of proinflammatory cytokines and chemokines [interleukin-6 (IL-6), IL-8, interferon gamma-induced protein 10 (IP-10), and monocyte chemoattractant protein-1 (MCP-1)], which were associated with progression to ARDS. Counterintuitively, neutralizing antibody responses were higher in patients who succumbed compared to those that recovered (*12*). A similar phenomenon has been described with the H1N1 2009 influenza pandemic where preexisting antibodies were associated with greater disease severity (*13*). Macaque studies of SARS-CoV-1 infection demonstrated that antibodies targeting the viral spike protein promoted inflammatory responses, recruitment of monocytes/macrophages to the lung, and disrupted wound healing (*12*). Incubation of monocyte-derived macrophages with immune serum from deceased, but not recovered patients with SARS, resulted in increased IL-8 production. The activation of macrophages was abrogated when experiments were conducted in the presence of Fc gamma receptor (FcγR)–blocking antibodies, suggesting that it occurred as a result of Fc receptor binding (*12*).

To understand whether these or other mechanisms were occurring in patients with SARS-CoV-2 infection, and in a subset of children with MIS-C, we compared humoral and cellular immunologic responses in pediatric versus adult hospitalized patients with COVID-19 to understand the relationships between immune responses, age, and clinical course.

## RESULTS

### Clinical outcomes in children and youth differ from those of adults

We compared demographics, clinical characteristics, and outcomes among children and youth (age <24 years, *n* = 65) and adults (>24 years, *n* = 60) with COVID-19 (Table 1), who were hospitalized at the Montefiore Medical Center in the Bronx, New York City, between 13 March and 17 May 2020. On the basis of WHO and United Nations designations of youth, the U.S. Centers for Disease Control and Prevention (CDC) definition of individuals <21 years as having MIS-C, and the age distribution of our cohort, we defined the pediatric patients as age <24 years (*14*, *15*). There were only two patients between the ages of 21 and 30, both were 22 years old.

**Table 1:** Demographics and clinical features of children and adults with COVID-19. Results are presented as means ± SD. *n*, number; COPD, chronic obstructive pulmonary disease.

	Age <24 (*n* = 65)*	**Age >24 (*n* = 60)**	***P* value^†^**
Age	13.34 ± 6.09	61.05 ± 12.96	<0.0001
Male:female (*n*)	41:24	34:26	0.47
Black:white:other/unknown (*n*)	25:5:35	30:7:23	0.22
Hispanic (*n*, %)	26 (40%)	15 (25%)	0.09
BMI	27.19 ± 14.09	29.78 ± 5.61	0.198
**Underlying medical conditions (*n*)**			
Obesity (BMI > 30)	18	21	0.44
Diabetes mellitus	8	20	0.0056
Asthma or COPD	18	12	0.40
Hypertension	3	35	<0.0001
**Treatment (*n*)**			
Hydroxychloroquine	9	47	<0.0001
Remdesivir	8	4	0.37
Systemic corticosteroid	14	8	0.25
IVIG	10	0	<0.0001
Other biologics^‡^	4	5	>0.99
**Outcome**			
LOS (days)^§^	6.37 ± 5.91	14.77 ± 16.68	<0.0001
Mechanical ventilation (*n*, %)	5 (7.7%)	22 (36.7%)	<0.0001
Deaths (*n*, %)	2 (3.1%)	17 (28.3%)	0.0001

*Includes 20 patients with MIS-C.

†Continuous variables were compared by Student’s *t* test; categorical variables were compared by Fisher’s exact test.

‡Other biologics include tocilizumab, sarilumab, and anakinra.

§LOS excludes patients who died.

Adults were more likely to have diabetes and hypertension compared to pediatric patients (Table 1). The adult patients were also more likely to have received hydroxychloroquine, whereas pediatric patients were more likely to have received intravenous immunoglobulin (IVIG). The latter was prescribed primarily for pediatric patients with MIS-C (*n* = 10/20). The length of stay (LOS) was significantly shorter in pediatric patients (including the 20 with MIS-C) compared with adults (*P* < 0.0001) (Table 1). Moreover, 22 adults (37%) required mechanical ventilation compared to only 5 (8%) of the pediatric patients (*P* < 0.0001). The in-hospital mortality rates were also significantly different with 17 adult (28%) compared to 2 pediatric (3%) deaths (*P* = 0.0001); none of the pediatric patients with MIS-C died (Table 1). The majority of deaths (86%) occurred in patients between the ages of 60 and 80 years.

### Clinical characteristics and serum cytokine concentrations by patient group

Because the presentations and outcomes in patients were heterogeneous, the patients were further subdivided into five groups (Table 2). These groups included pediatric patients with acute presentations (typically fever, respiratory, or gastrointestinal symptoms), who did not require mechanical ventilation (group 1, *n* = 41, LOS = 4.84 ± 5.36 days, mean ± SD), pediatric patients with MIS-C (group 2, *n* = 20, LOS = 8.1 ± 4.05), adults who recovered and did not require mechanical ventilation (group 3, *n* = 33, LOS = 7.88 ± 6.84 days), and adults who required mechanical ventilation or died (group 4, *n* = 27, LOS = 37.50 ± 19.60 days excluding those who died; *P* < 0.0001 versus groups 1, 2, and 3). There were also four non–MIS-C pediatric patients who developed progressive respiratory disease requiring mechanical ventilation (ages 14, 18, 19, and 22) (group 5), two of whom died (median LOS for the two surviving patients 21.0 ± 9.90 days) (Table 2). The patients with MIS-C (group 2) had a significantly lower body mass index (BMI) compared to patients in the other groups, but there was no significant difference in BMI between groups 1, 3, 4, and 5 (Table 2).

**Table 2:** Clinical and laboratory findings in subgroups of pediatric and adult patients with COVID-19. Data are mean ± SD. ANC, absolute neutrophil count; Gp, group; WBC, white blood cell; NS, not significant.

	**Group 1**	**Group 2**	**Group 3**	**Group 4**	**Group 5**	*P* value^*^
	**Age <24 (no** **mechanical** **ventilation)** **(*n* = 41)**	**MIS-C (*n* = 20)**	**Adult (no** **mechanical** **ventilation)** **(*n* = 33)**	**Adult (mechanical** **ventilation or** **death) (*n* = 27)**	**Age <24** **(mechanical** **ventilation or** **death) (n = 4)**	
Age (years)	14.90 ± 5.61	9.15 ± 5.28	59.42 ± 14.89	63.04 ± 10.05	18.25 ± 3.30	<0.0001
Days since onset of symptoms	4.51 ± 4.57 (*n* = 33)	4.06 ± 1.47 (*n* = 18)	5.00 ± 3.04 (*n* = 31)	4.70 ± 2.91 (*n* = 23)	3.75 ± 2.75 (*n* = 4)	NS
BMI	30.45 ± 15.48 (*n* = 36)	19.04 ± 5.81 (*n* = 19)	29.61 ± 5.77 (*n* = 31)	29.99 ± 5.52 (*n* = 26)	28.00 ± 9.93 (*n* = 4)	<0.01 (Gp 2 versus 1, 3, and 4) and <0.05 (Gp 2 versus 5)
LOS^†^	4.84 ± 5.36 (*n* = 38)	8.10 ± 4.05 (*n* = 20)	7.88 ± 6.84 (*n* = 33)	37.50 ± 19.60 (*n* = 10)	21.00 ± 9.90 (*n* = 2)	<0.0001 (Gp 4 versus 1, 2, and 3)
Mortality (*n*)	0	0	0	17	2	<0.0001 (Gp 4 versus 1, 2, and 3)
WBC (×10^−3^ cells/μl)	9.89 ± 6.63	9.39 ± 3.78	7.15 ± 4.57	8.29 ± 2.62	11.15 ± 4.01	NS
Hemoglobin (g/dl)	13.22 ± 2.86	11.23 ± 1.83	12.65 ± 2.02	12.89 ± 2.32	12.33 ± 1.24	NS
Platelets (×10^−3^ cells/μl)	265.29 ± 144.25	174.95 ± 98.23	206.62 ± 80.29	214.85 ± 823.28	181.25.00 ± 538.84	NS
ANC (cells/μl)	6878.05 ± 5664.74	7750.00 ± 3623.53	5339.39 ± 4224.77	6533.33 ± 2344.55	8900.00 ± 2892.52	NS
ALC (cells/μl)	2092.68 ± 1443.50	1105.00 ± 592.47	1069.70 ± 470.69	1129.63 ± 685.46	1450.00 ± 925.56	<0.01 (Gp 1 versus 2, 3, and 4)
CRP (mg/dl)	4.56 ± 7.11 (*n* = 34)	18.80 ± 13.57 (*n* = 19)	12.83 ± 7.75 (*n* = 20)	19.50 ± 12.83 (*n* = 17)	18.95 ± 11.37 (*n* = 4)	<0.0001 (Gp 1 versus 2 and 4) and <−0.05 (Gp 1 versus 3)
CRP (peak) (mg/dl)	9.66 ± 10.67 (*n* = 31)	24.49 ± 10.95 (*n* = 18)	14.10 ± 8.90 (*n* = 25)	31.06 ± 17.94 (*n* = 23)	24.13 ± 9.98 (*n* = 4)	<0.0001 (Gp 1 and 3 versus 4) and <0.01 (Gp 1 versus 2)
Ferritin (peak) (ng/ml)	969.52 ± 1155.33 (*n* = 27)	1893.16 ± 3144.32 (*n* = 19)	1276.11 ± 1551.25 (*n* = 9)	4158.47 ± 5192.46 (*n* = 17)	1257.25 ± 1076.43 (*n* = 4)	NS
LDH (peak) (U/liter)	415.78 ± 255.56 (*n* = 27)	509.74 ± 434.86 (*n* = 19)	460.00 ± 165.62 (*n* = 26)	770.88 ± 553.94 (*n* = 24)	797.25 ± 233.62 (*n* = 4)	<0.01 (Gp 1 versus 4) and <0.05 (Gp 3 versus 4)
Troponin (peak) (ng/ml)	0.01 ± 0.005 (*n* = 25)	0.09 ± 0.16 (*n* = 18)	0.12 ± 0.56 (*n* = 30)	0.07 ± 0.10 (*n* = 26)	0.10 ± 0.12 (*n* = 4)	NS
CPK (peak) (U/liter)	645.65 ± 1150.71 (*n* = 20)	517.47 ± 438.42 (*n* = 17)	537.93 ± 675.00 (*n* = 29)	757.29 ± 1307.40 (*n* = 24)	1088.25 ± 690.81 (*n* = 4)	NS
D-dimer (peak) (μg/ml)	1.43 ± 1.52 (*n* = 25)	11.14 ± 7.00 (*n* = 18)	2.58 ± 1.72 (*n* = 17)	12.57 ± 9.46 (*n* = 17)	9.30 ± 5.96 (*n* = 4)	<0.001 (Gp1 versus 2; 2 versus 3), <0.01 (Gp 1 versus 4), and <0.05 (Gp 3 versus 4)

*Groups 1 to 4 were compared by ANOVA with Tukey’s multiple comparison.

†Laboratory values are from the time of admission unless indicated as peak.

**Fig. 1:**
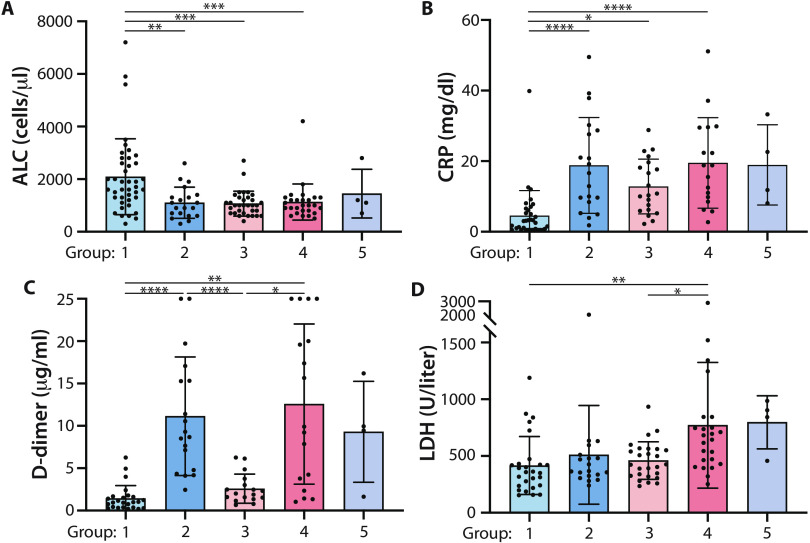
Clinical characteristics vary by age and patient outcome. Shown are clinical measurements at hospital admission for pediatric patients who did not require ventilation (group 1), patients with MIS-C (group 2), adults who did not require ventilation (group 3), adults who required mechanical ventilation or died (group 4), and pediatric patients who required mechanical ventilation (group 5). Clinical measurements included absolute lymphocyte counts (ALC) (**A**) and serum concentrations of CRP (**B**), peak D-dimer (**C**), and peak LDH (**D**). Data are presented as mean ± SD and compared by one-way ANOVA with Tukey post hoc comparisons. *P < 0.05, **P < 0.01, ***P < 0.001, and ****P < 0.0001. (ALC, *n* = 41, 20, 33, 27, and 4 in groups 1 to 5; CRP, *n* = 34, 19, 20, 17, and 4 in groups 1 to 5; D-dimer, *n* = 25, 18, 17, 17, and 4 in groups 1 to 5; LDH, *n* = 27, 19, 26, 24, and 4 in groups 1 to 5).

The absolute lymphocyte counts were higher in the non–MIS-C pediatric group (group 1) compared to the MIS-C pediatric group (group 2) and compared to both adult groups [*P* < 0.01, analysis of variance (ANOVA)] (Fig. 1A and Table 2). Group 1 also had lower serum concentrations of C-reactive protein (CRP) and D-dimer compared to those in the MIS-C group (group 2) and compared to both adult groups (Fig. 1, B and C). D-dimer was also higher in ventilated compared to nonventilated adults (*P* < 0.05, group 4 versus group 3). The serum lactate dehydrogenase (LDH) concentration was also higher in group 4 compared to groups 1 and 3 (Fig. 1D). Other clinical laboratory values did not differ significantly between groups (Table 2).

**Fig. 2:**
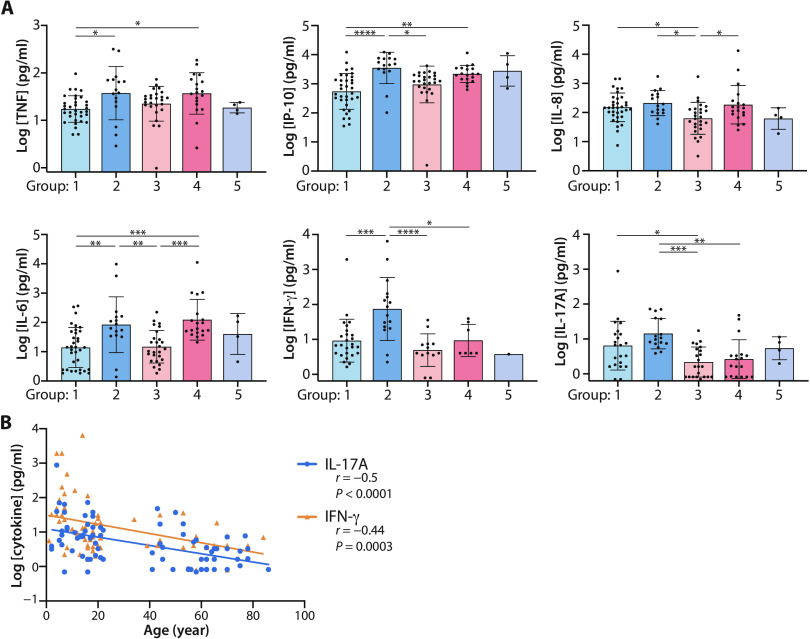
Serum cytokine concentrations vary by age and patient outcome. Cytokine concentrations in remnant serum samples obtained within 7 days of admission were determined using a multiplexed Luminex assay. (**A**) The concentrations in each group were compared by one-way ANOVA with Tukey’s post hoc comparison (mean ± SD; *P < 0.05, **P < 0.01, ***P < 0.001, and ****P < 0.0001). Sample sizes for groups 1 to 5 are as follows: TNF, *n* = 34, 16, 26, 19, and 4; IP-10, *n* = 34, 16, 26, 19, and 4; IL-8, *n* = 34, 16, 26, 19, and 4; IL-6, *n* = 34, 16, 26, 19, and 4; IFN-γ, *n* = 26, 16, 13, 7, and 1; IL-17A, *n* = 22, 16, 22, 18, and 4. (**B**) Correlation between serum concentrations and age for IL-17A (*n* = 82) and IFN-γ (*n* = 63) as determined by Spearman’s test.

Elevated serum concentrations of IL-6, tumor necrosis factor–α (TNF-α), and other inflammatory cytokines have been described in adults with COVID-19, particularly those who progress to ARDS (*16*). However, there is limited data in pediatric patients. We therefore compared cytokine concentrations in remnant serum samples obtained within 1 week of presentation. Patients in group 1 (non–MIS-C pediatric) had lower concentrations of IL-6, TNF-α, and IP-10 compared to the pediatric MIS-C group (group 2, *P <* 0.01, *P <* 0.05, and *P <* 0.0001, respectively) and compared to adults with more severe outcomes (group 4, *P <* 0.0001, *P <* 0.05, and *P <* 0.01, respectively; Tukey’s multiple comparison one-way ANOVA) (Fig. 2A). Patients in group 3 (nonventilated adults) also had lower concentrations of IL-6 compared to group 4 (*P <* 0.001) (Fig. 2A). Conversely, the concentration of IL-17A was higher in group 1 versus group 3 (*P <* 0.05) and in group 2 versus groups 3 and 4 (*P <* 0.0001 and *P <* 0.01, respectively). Similarly, interferon-γ (IFN-γ) concentrations were higher in group 2 compared to both adult groups (*P <* 0.001 and *P <* 0.01), as well as in comparison to group 1 (*P <* 0.001) (Fig. 2A).

These findings suggested that age may contribute to the higher concentrations of circulating IL-17A and IFN-γ in the younger patients, and thus, we examined the relationship between cytokine concentrations and age. There was a significant negative correlation between age and the concentrations of IL-17A (Spearman *r* = −0.50, *P <* 0.0001) and IFN-γ (*r* = −0.44, *P* = 0.0003), but not IL-6, TNF, or IP-10 (Fig. 2B). Recognizing that patients with MIS-C may have a delayed presentation relative to the time of SARS-CoV-2 infection, we also analyzed this relationship excluding patients with MIS-C. The significant negative correlation between IL-17A concentration and age was still seen (*r* = −0.38, *P <* 0.01).

### T cell responses to viral spike protein in patients with COVID-19

**Fig. 3:**
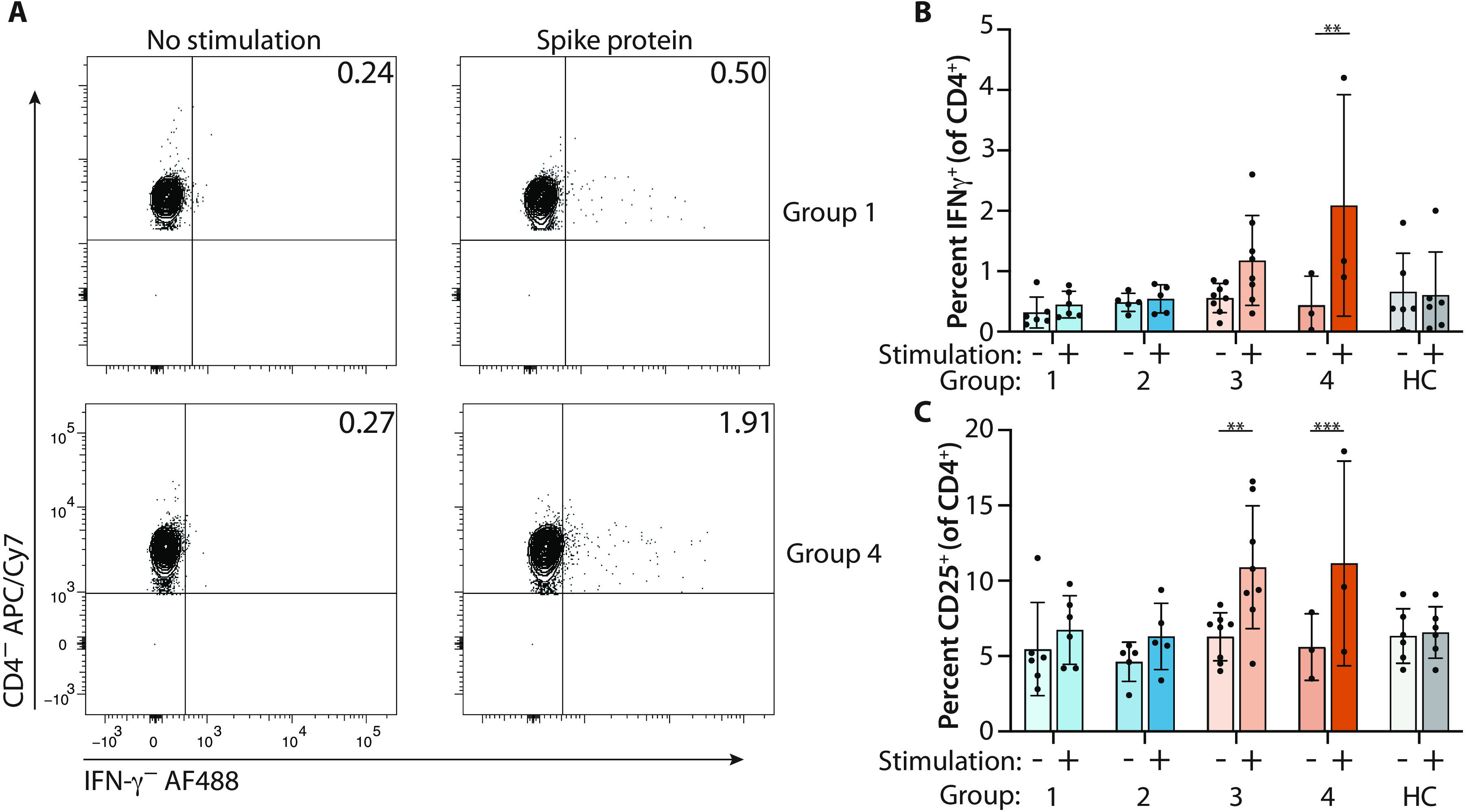
CD4^+^ T cell responses to SARS-CoV-2 spike protein. PBMCs from 22 patients [group 1, *n* = 6; group 2, *n* = 5; group 3, *n* = 8; group 4, *n* = 3; healthy control (HC), *n* = 6] were incubated with or without intact SARS-CoV-2 spike protein for 24 hours. The CD4^+^ T cells were analyzed for intracellular IFN-γ (**A** and **B**) and CD25 (**C**). Representative flow cytometry plots of intracellular IFN-γ staining in CD4^+^ T cells before and after stimulation with spike protein are shown in (A). The data were analyzed by multiple regression with a mixed model for repeated measures with comparisons with and without spike protein (mean ± SD; **P < 0.01 and ***P < 0.001).

We next compared cellular immune responses by culturing peripheral blood mononuclear cells (PBMCs) from blood samples from adult and pediatric patients with COVID-19 for 24 hours with the intact viral spike protein and quantifying intracellular cytokine staining as well as CD25 expression on CD4^+^ T cells. The PBMCs were harvested from 22 patients 40 ± 9.6 days after hospitalization (group 1, *n* = 6; group 2, *n* = 5; group 3, *n* = 8; group 4, *n* = 3; healthy control, *n* = 6). There was a significant difference in the induction of IFN-γ^+^ in CD4^+^ T cells between the groups (*P* = 0.04, ANOVA) (Fig. 3, A and B). We did not identify differences in the frequency of IL-17A^+^ or TNFα^+^ CD4^+^ T cells. Consistent with the findings of intracellular IFN-γ production, there was also a difference in the induction of CD25 (*P* = 0.014, ANOVA). We found higher expression of CD25 on CD4^+^ T cells in patients in groups 3 and 4 (adult), but not in groups 1 and 2 (pediatric), after stimulation with spike protein (group 3, *P* = 0.0002; group 4, *P* = 0.0059; Sidak’s multiple comparison) (Fig. 3C). This suggested a more robust T cell response to the spike protein in the adult compared to the pediatric patients.

### Immunoglobulin responses in patients with COVID-19

**Fig. 4:**
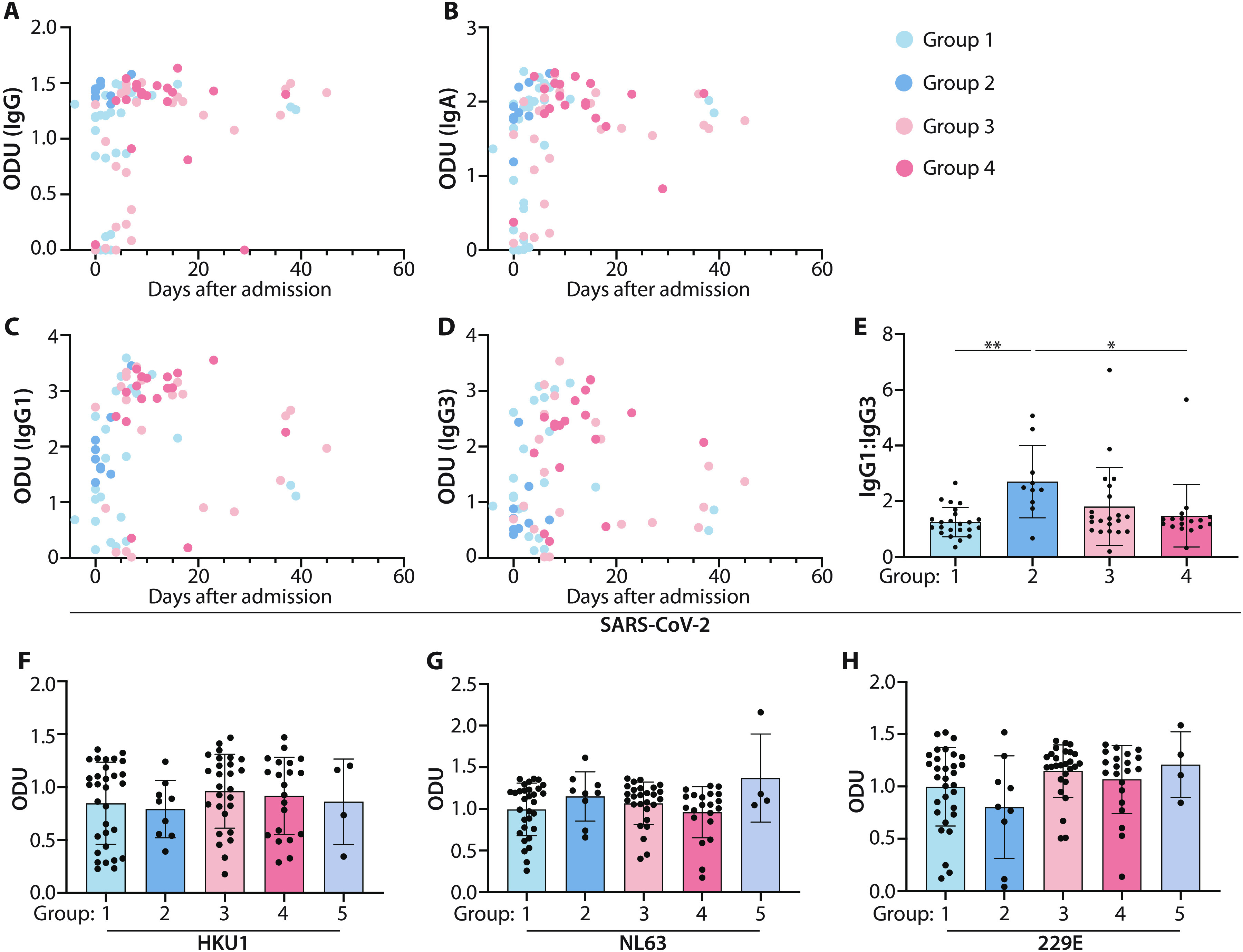
Spike protein–specific antibody titers in patient serum samples. Anti–SARS-CoV-2 spike protein total IgG (**A**), IgA (**B**), IgG1 (**C**), and IgG3 (**D**) were measured by ELISA at a 1:50 serum dilution. Data are presented as ODUs in relation to the time serum was obtained after admission (*n* = 90) for IgG (Spearman *r* = 0.35, *P* = 0.0008), IgA (*r* = 0.35, *P* = 0.0008), IgG1 (*r* = 0.31, *P* = 0.01), and IgG3 (*r* = 0.24, *P* = 0.047). (**E**) Ratio of anti–SARS-CoV-2 spike protein–specific IgG1 to IgG3 in 71 patients with detectable IgG (group 1, *n* = 23; group 2, *n* = 10; group 3, *n* = 21; and group 4, *n* = 17) (*P < 0.05, **P < 0.01, and ***P < 0.0001, ANOVA with Tukey multiple comparison test). (**F** to **H**) Shown are serum IgG antibodies to other human CoVs: (F) HKU1, (G) NL63, and (H) 229E. In (F) to (H), *n* = 33, 9, 26, 22, and 3 in groups 1 to 5.

Concentrations of anti–SARS-CoV-2 spike protein immunoglobulin A (IgA) and total and subclass IgG antibodies were measured in remnant serum samples (*n* = 90). Overall, there was a significant relationship between the time from admission when the serum samples were obtained and the concentration of anti-spike protein IgG (Spearman *r* = 0.35, *P* = 0.0008), IgA (*r* = 0.35, *P* = 0.0008), IgG1 (*r* = 0.31, *P* = 0.01), and IgG3 (*r* = 0.24, *P* = 0.047) (Fig. 4, A to D). The anti-spike protein IgG2 and IgG4 antibodies were below the limit of detection in most samples. The time from admission for sample collection was shorter in group 1 (5.18 ± 9.22 days, *n* = 34) compared to group 3 (12.7 ± 12.86 days, *n* = 27) or group 4 (12.89 ± 8.97 days, *n* = 19) (*P* = 0.019 and *P* = 0.03, respectively, ANOVA with Tukey’s multiple comparison). The patients with MIS-C (group 2) were presumably exposed to the virus earlier in relation to the time of admission, and their serum samples were obtained 1.67 ± 2.35 days after admission. To assess potential between-group differences in IgG subclass antibodies, we compared the ratio of IgG1 to IgG3 in a total of 71 patients with detectable IgG (group 1, *n* = 23; group 2, *n* = 10; group 3, *n* = 21; and group 4, *n* = 17). The ratio differed significantly between the groups (*P* = 0.0075, ANOVA). The ratio was higher in patients with MIS-C (group 2) (2.70 ± 1.3) compared to group 1 (1.26 ± 0.53, *P* = 0.0048), group 3 (1.81 ± 1.4, *P* = 0.16), and group 4 (1.48 ± 1.12, *P* = 0.03) (ANOVA with Tukey’s multiple comparison; Fig. 4E). We also compared antibody titers to other common cold human CoVs (229E, NL63, and HKU1) and found no significant differences across the groups (Fig. 4, F to H).

**Fig. 5:**
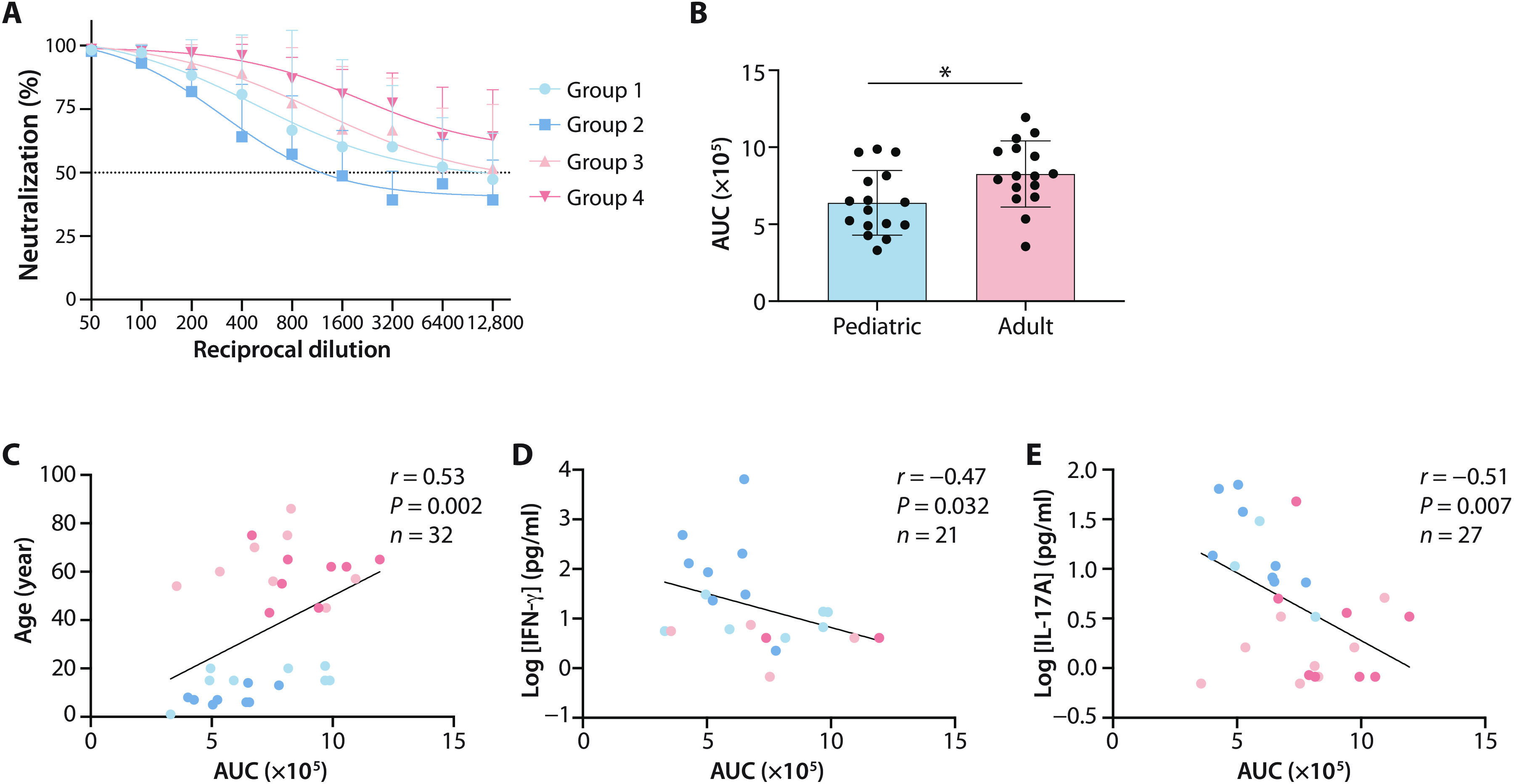
Neutralizing antibody titers in patient serum vary by age. (**A**) VSV-S was incubated with serial twofold dilutions of patient serum or culture media as a control for 1 hour at 37°C and subsequently was added to cultured Vero cell monolayers. Neutralization of VSV-S by antibody was measured after 48 hours by comparing reduction in plaque number relative to control wells. (**B**) A comparison of AUC for neutralizing antibody data in (A) for pediatric and adult patients. (**C** to **E**) Correlations between neutralizing antibody AUC and age in years (C) or serum concentrations of IFN-γ (D) and IL-17A (E) and age in years are presented. In (A), *n* = 8 per group; in (B), *n* = 16 per group. Data in (A) and (B) are presented as mean ± SD. Data in (B) were analyzed by unpaired Student’s *t* test. Correlations in (D) to (**F**) were determined by Spearman’s nonparametric correlation. *P < 0.05.

Between-group differences in the functionality of the anti-spike protein IgG antibodies were also evaluated. For these studies, we selected a subset of the above serum samples (*n* = 8 per group) with comparable total anti-spike protein IgG [mean optical density units (ODUs) 1.46 ± 0.06] (Fig. 5A). Dose-response curves showed significant between-group differences in the ability of serum to neutralize a recombinant vesicular stomatitis virus expressing the SARS-CoV-2 spike protein (VSV-S) (*P* = 0.037, two-way ANOVA). Significantly lower neutralizing activity was detected in the combined pediatric (groups 1 and 2) compared to adult (groups 3 and 4) cohorts [*P* = 0.019, area under the curve (AUC)] (Fig. 5B). There were three patients in group 4 and one each in groups 1 and 3 that had neutralizing titers greater than 12,800. The neutralization activity correlated positively with age (*P* = 0.002) and negatively with IFN-γ (*P* = 0.032) and IL-17A (*P* = 0.005) concentrations (Fig. 5, C to E).

**Fig. 6:**
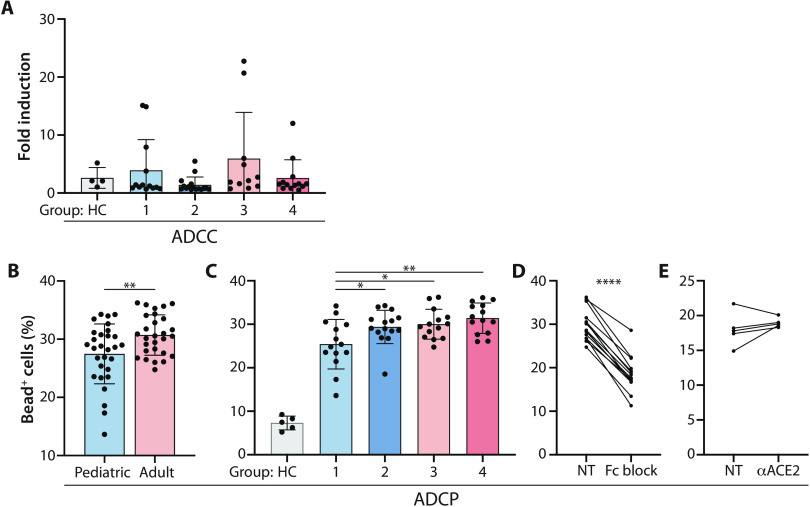
Antibody effector functions vary by clinical outcome. (**A**) ADCC activity of serum antibody was measured by an FcγRIIIa bioreporter assay and expressed as fold induction. (**B** and **C**) ADCP was measured as the percent of THP-1 cells internalizing spike protein–coated beads in the presence of COVID-19 patient serum after 16 hours of culture. ADCP was compared between pediatric and adult patient serum samples (B) and by group (C). (**D** and **E**) ADCP after (D) an Fc-blocking antibody (2 μg per sample) or (E) an ACE2-blocking antibody (2 μg/ml) was added to a subset of serum samples to assess the role of Fc receptors and ACE2, respectively, in bead internalization by THP-1 cells. HC, healthy control sera obtained before 2020. NT, no treatment. In (B), *P <* 0.0001 for comparisons between healthy control serum samples and all other groups. Statistics were calculated by unpaired Student’s *t* test (B), one-way ANOVA (C), or paired Student’s *t* test (D). *P < 0.05, **P < 0.01, and ****P < 0.0001. Data are presented as mean ± SD (A and B). [(A), *n* = 4, 13, 16, 11, and 13 in HC and groups 1 to 4; (B), *n* = 29 and 28 for pediatric and adult serum samples, respectively; (C), *n* = 5, 14, 15, 14, and 14 in HC and groups 1 to 4; (D), *n* = 15; (E), *n* = 5].

We then measured non-neutralizing antibody-dependent cellular cytotoxicity (ADCC) and antibody-dependent cellular phagocytosis (ADCP) activity in the same serum samples using spike protein–coated microspheres as the targets. There were no significant differences in ADCC activity across the groups, although a few patients had ADCC activity greater than observed with serum obtained from healthy volunteers before the COVID-19 pandemic (Fig. 6A). In contrast, increased phagocytic activity was detected when spike protein–coated beads were incubated with serum from patients with COVID-19 compared to controls (*P <* 0.0001). The phagocytic activity was significantly lower when comparing combined pediatric (groups 1 and 2) versus adult (groups 3 and 4) patients (*P <* 0.01) (Fig. 6B). Group 1 exhibited the lowest phagocytic activity compared to the other groups (*P <* 0.01) (Fig. 6C). The uptake of beads was reduced in the presence of Fc blockade (Fig. 6D), but not in the presence of a polyclonal antibody to angiotensin-coverting enzyme 2 (ACE2) (Fig. 6E), suggesting that bead uptake was Fc receptor mediated.

## DISCUSSION

The clinical manifestations and outcomes from COVID-19 differ by age as evidenced by a shorter length of hospital stay, decreased requirement for mechanical ventilation, and decreased mortality in children (including those who presented with MIS-C) compared to adults. Results of our study indicate that the poorer outcomes in adults were not attributable to a failure to generate T cell or antibody responses to the virus. The opposite was observed as adults had higher neutralizing antibody titers, ADCP, and more vigorous T cell responses to viral spike proteins compared to pediatric patients. Immunity to other common cold human CoVs has been speculated to provide cross-protection or to promote antibody-mediated enhancement (*17*). However, we did not identify differences in preexisting antibodies to other human CoVs by age or outcome, suggesting that they do not play a dominant role in modulating the clinical response.

Further dissection of the immune responses identified several differences that may provide insights into how host responses contribute to clinical outcomes. Specifically, pediatric patients had higher serum concentrations of IL-17A and IFN-γ shortly after presentation. This age-associated difference was most notable for IL-17A, which persisted even after excluding the patients with MIS-C who were delayed in their hospitalization from the initial SARS-CoV-2 exposure compared to patients in the other groups. This observation suggests that IL-17A or the cells that produce it may contribute to immune protection, particularly against lung disease, which was milder in group 1 and an uncommon manifestation of MIS-C. IL-17A is produced by multiple cell types including CD4^+^ T cells, CD8^+^ T cells, γd T cells, invariant natural killer T (NKT) cells, innate lymphoid cells, and neutrophils. We did not find a robust IL-17A response, measured by intracellular cytokine staining in CD4^+^ T cells, at times when we could detect intracellular IFN-γ (Fig. 3), suggesting that other cells may be the primary source of IL-17A in serum. Likewise, the frequencies of IFN-γ^+^ CD4^+^ T cells were highest in samples obtained from the adult patients (groups 3 and 4), suggesting that other cells may be the source of this cytokine in the serum. Our findings are consistent with a recent report in which IFN-γ, but not IL-17A, was detected in T cells from patients with COVID-19 that were stimulated with peptide from the virus (*18*).

Several preclinical and clinical studies of bronchoalveolar lavage (BAL) samples from patients with other respiratory infections and diseases have identified tissue-resident immune cells that may produce IL-17A and IFN-γ, although the specific role of these cytokines is not clear (*19*). Dual cytokine (IL-17A and IFN-γ)–producing resident memory cells have been described in the lung (*19*), and a protective role for T helper 17 cells has been described in pulmonary infections (*20*). In addition, pulmonary epithelial cells have been shown to be a source of IFN-γ during infection with *Mycobacterium tuberculosis* (*21*). Possibly, the higher concentrations of IFN-γ and IL-17A in the serum of pediatric patients with COVID-19 reflected increased expression by cells in the respiratory tract, and these local cytokines may have protected the patients from progressive respiratory disease. The cytokines may have contributed to more rapid clearance of the virus, although we did not obtain quantitative SARS-CoV-2 polymerase chain reaction (PCR) data, longitudinal nasopharyngeal swabs, or BAL samples to test this directly. Virus clearance is suggested by the observation that half of the patients with MIS-C did not have viral RNA detected in their nasal swabs at the time of presentation.

The age-related difference in IL-17A concentrations in serum is consistent with described dysfunction of innate immune responses in older individuals (*22*). Decreased expression of pattern recognition receptors such as retinoic-acid inducible gene-1 (RIG-I) by monocytes has been found with aging and has been postulated to account for reduced type 1 IFN release. Invariant NKT cells, which secrete IL-17A and have cytolytic function, also decrease in number and function with aging (*23*).

A consequence of a more robust innate immune response in children might be a diminished adaptive immune response. This notion is supported by the observation of a lower frequency of antigen-reactive (spike protein) CD25^+^ and IFN-γ–producing CD4^+^ T cells after stimulation with spike protein, lower neutralizing antibody titers, and less ADCP activity in pediatric compared to adult patients, and an inverse correlation between neutralizing antibody titer and serum concentrations of IL-17A and IFN-γ. Group 1 pediatric patients with COVID-19 who recovered without sequelae exhibited the lowest ADCP activity and had the lowest serum concentrations of IL-6 and TNF, cytokines associated with ARDS, and poor outcome in clinical studies (*16*). These observations raise the possibility that release of cytokines by Fc receptor–bearing cells during phagocytosis may contribute to the inflammatory cytokine storm linked to progressive ARDS in adults or MIS-C in children (*24*, *25*). The notion that ADCP may contribute to enhanced pathology has been suggested in previous studies with other viruses, most notably dengue virus and the related flavivirus, Zika virus (*26*, *27*). The findings are also reminiscent of earlier studies in adults with SARS-CoV-1 infection, where both neutralizing antibody and ADCP responses were higher in patients who died compared to those who recovered (*12*). As with the current studies, Fc receptor blockade inhibited the response, indicating that the phagocytic activity was mediated by interactions between the antibodies and Fc receptors.

Whereas neither the quantity nor functionality of antibody responses differed when comparing the adults by outcome, differences were observed between the pediatric cohorts. Specifically, the pediatric patients with MIS-C (group 2) had a greater proportion of IgG1 versus IgG3 spike-specific antibodies and more ADCP activity compared to non–MIS-C pediatric patients. Differences in antibody function may be due to subclass, Fc glycans, and antigenic targets. The differences in the relative proportion of IgG1 and IgG3 in patients with MIS-C may also contribute to the differences in immune responses directed at other viral proteins including membrane or nucleocapsid proteins, which were not evaluated in the current study.

The results from our study provide new insights into potential mechanisms that may contribute to age-related differences in disease resolution or enhanced pathology in patients with COVID-19 and may have implications for ongoing efforts with convalescent plasma and the development of therapeutic antibodies. The finding that adults who did poorly had high titers of antibodies that were both neutralizing and induced phagocytosis, as well as the greater T cell responses suggest that boosting of these functional responses to the spike protein, particularly late in the course of disease, may not be beneficial. In contrast, boosting of early innate immune responses may be important.

There are a number of limitations to our study. The patients and their immunologic measurements were heterogeneous, and therefore, differences could be missed because of the variance within the groups and the small sample size. Some of the patients received brief treatment with hydroxychloroquine, remdesivir, methylprednisolone, IVIG, or other therapies during the course of their hospitalization, and we cannot exclude an effect of these agents on cellular immune responses. In addition, we did not have access to BAL or tissue from the patients, and there may be findings in the lung or regional lymph nodes that are not reflected in the peripheral blood. Last, we did not have access to serial samples to study the kinetics of the immune responses or the release of other mediators such as type 1 IFNs. We cannot track the exact time of exposure to SARS-CoV-2, and we did not have results for viral shedding so there may have been differences in immune responses during the clinical course that went undetected. Our assessment of progression through the disease course could only be assessed clinically.

In summary, we have identified differences in humoral and cellular immune responses to SARS-CoV-2 infection between pediatric and adult patients with COVID-19 and have distinguished responses that were age related or associated with the clinical course. Our studies suggest that early immune responses mediated by cells producing IL-17A and IFN-γ resulted in more rapid resolution of the viral infection and may have mitigated against the progressive cytokine release and tissue pathology that occurs with more robust adaptive immune responses.

## MATERIALS AND METHODS

### Study design

The goal of this study was to compare clinical characteristics and cellular and humoral immune responses in children and adults who were hospitalized at the Montefiore Medical Center with confirmed SARS-CoV-2 infection (PCR assay or positive serology). Serum and PBMCs were obtained from a total of 125 patients who were admitted to the Montefiore Medical Center between 13 March and 31 May 2020. This study was approved by the Institutional Review Board of the Albert Einstein College of Medicine (IRB no. 2020-11278). Patients were excluded if they had preexisting medical conditions that might affect immune responses including cancer and HIV or were receiving chronic immunosuppressive therapy for transplantation or other conditions. Remnant serum samples were obtained from the Montefiore Clinical Laboratory. Sera were divided into aliquots and stored at −80°C. After obtaining informed consent (and assent from pediatric patients), PBMCs were isolated from a subset of patients (Cell Preparation Tubes, BD Biosciences). Clinical data were extracted from the electronic medical record. MIS-C was defined by CDC criteria (*15*). De-identified samples obtained before 2020 and available from a biorepository of HIV seronegative sera and PBMCs isolated from leukopaks (New York Blood Bank) were included as healthy control samples. Laboratory measurements (complete blood counts, blood chemistries, ferritin, D-dimer, and CRP) were measured in the clinical laboratory at the Montefiore Medical Center, Bronx, New York City.

### Cytokine measurements

Cytokine concentrations in sera and supernatants from the cultures of PBMCs were measured using an 11-plex Milliplex MAP Human Cytokine/Chemokine Magnetic Bead Panel (Millipore). Cell culture supernatants were harvested at 24 hours. Samples were plated and prepared per the manufacturer’s instructions. Data were acquired on a Luminex Magpix (Luminex Corporation) and analyzed in the Milliplex Analyst program (Millipore). Results below the lower limit of detection (LLOD) were set at the LLOD.

### Enzyme-linked immunosorbent assay

Recombinant SARS-Cov-2 spike protein (ACROBiosystems, #S1N-C52H3), HKU1 S1 protein (Sino Biological, #40021-V08H-100), 229E S1 protein (Sino Biological, #40601-V08H-100), or NL63 S1 protein (Sino Biological, # 40600-V08H-100) at a concentration of 100 ng in 50 μl of phosphate-buffered saline (PBS) were added to 96-well MaxiSorp plates (Thermo Fisher Scientific, #442404) and incubated overnight at 4°C. Plates were washed three times with 250 μl of enzyme-linked immunosorbent assay (ELISA) wash buffer PBS-T (PBS with 0.1% Tween 20) and incubated with 200 μl of blocking solution (PBS with 0.1% Tween 20 and 3% milk powder) at room temperature (RT) for 1.5 hours. A 1:50 dilution of individual serum samples was added to wells and allowed to bind for 2 hours at RT. Wells were washed three times with PBS-T and then incubated with horseradish peroxidase–conjugated anti-human IgG (1:5000 dilution; GenScript, #A00166), anti-human IgG isotype specific (Southern Biotech; IgG1, clone HP6001; IgG2, clone 31-7-4; IgG3, clone HP6050; and IgG4, clone HP6025), or anti-human IgA antibody (1:2000 dilution; BioLegend, #411002) for 1 hour at RT. Plates were washed six times with PBS-T and developed with tetramethylbenzidine (TMB) substrate (BD Biosciences, #55214) for 12 min. The reaction was stopped with 2 N H_2_SO_4_. The ODUs were measured on a Synergy HT BioTek plate reader (Winooski, VT), and ODUs from seronegative controls were subtracted from patient samples.

### Neutralization assay

Serial twofold dilutions of heat-inactivated serum (1:50 to 1:12800) were incubated with recombinant VSV-S (*28*) for 1 hour at 37°C. The inoculum was then added in duplicate to 24-well plates containing monolayers of Vero [African green monkey kidney; American Type Culture Collection (ATCC), CCL-81] cells for 1 hour at 37°C. After incubation, the inoculum was aspirated, cells overlaid with methylcellulose, further incubated for 48 hours at 37°C, and subsequently fixed and stained with crystal violet.

### Preparation of spike protein–coated microspheres

The extracellular domain of SARS-CoV-2 spike protein (amino acids 1 to 1208) containing the stabilizing proline mutations K986P and K987P, the furin site mutation RRAR:GSAS, a T4 trimerization domain, HRVC2 protease site, 8XHIS, and Twin-strep-tag on the C terminus was purified using nickel affinity chromatography from ExpiCHO-S cells as previously described (endotoxin, <0.02 EU/ml) (*29*) and biotinylated using the EZ-Link Micro Sulfo-NHS-SS-Biotinylation Kit (Thermo Fisher Scientific, #21945). Fluorescent NeutrAvidin microspheres (Invitrogen, F8775) were washed twice in PBS/0.1% bovine serum albumin (BSA) and incubated with biotinylated protein at a ratio of 10 μg of protein per 5 μl of beads. The final volume was brought to 200 μl in PBS/BSA and incubated overnight on a rotator at 4°C. Beads were washed to remove unbound protein.

### Antibody-dependent cell-mediated cytotoxicity

Spike protein microspheres were incubated with heat-inactivated sera (1:5 dilution) for 15 min at RT in white, flat-bottomed 96-well plates. Reporter cells expressing human FcγRIIIa V variant (Promega G7015) were added for 6 hours at 37°C, 5% CO_2_. FcγRIIIa activation was detected by the addition of luciferin substrate. Plates were read in a SpectraMax M5^e^ (Molecular Devices). Fold induction was calculated relative to luciferase activity in the absence of serum.

### Antibody-dependent phagocytosis

Ten microliters of diluted heat-inactivated serum was incubated with an equal volume of spike protein–coated fluorescent beads for 2 hours at 37°C, 5% CO_2_ to allow immune complexes to form. THP-1 (ATCC, TIB-202) cells were then added (5 × 10^4^ cells per well in a volume of 200 μl) and incubated for 16 hours at 37°C, 5% CO_2_. One-hundred microliters of supernatant was removed from each well and replaced with an equal volume of 4% paraformaldehyde and incubated for 20 min at RT. Cells were pelleted and washed once in fluorescence-activated cell sorting (FACS) buffer (10% fetal bovine serum and 0.5 mM EDTA in PBS), and percentage of THP-1 cells with internalized spike protein beads was quantified using a Cytek Aurora flow cytometer (Cytek Biosciences, Fremont, CA) and analyzed in FlowJo (BD Biosciences, Ashland, OR). Human Fc receptor–blocking agent (BD Pharmingen, 564220; 2 μg per well) and anti-human ACE-2 R antibody [R&D, AF933-SP; final concentration (2 μg/ml)] were added to THP-1 cells as additional controls.

### Antigen responses of PBMCs

PBMC from healthy donors (*n* = 6) (collected before 2020) and patients with COVID-19 who recovered (*n* = 22) were thawed and resuspended at 1 × 10^7^ cells/ml in RPMI supplemented with 5% human serum. One-hundred microliters of cell suspension (1 × 10^6^ cells) was transferred to wells of a 96-well U-bottom plate containing purified SARS-CoV-2 spike protein (40 μg/ml) or media alone. After 24 hours in a 37°C/5% CO_2_ incubator, GolgiStop (BD Biosciences, catalog no. 554724) was added for 6 hours. The cells were harvested, fixed, and permeabilized (BD, Phosflow FIX I, catalog no. 557870; Phosflow Perm III, catalog no. 558050) and stained with antibodies to CD3 (BV650) (clone OKT3, catalog no. 317323), CD4 (allophycocyanina (APC)/Fire750) (clone A161A1, catalog no. 357425), CD8 (phycoerythrin (PE)/Cy7) (clone SK1, catalog no. 344711), CD25 APC-CD25 (clone MA-251, catalog no. 356110), IL-17A (PE Dazzle 594, BL168, catalog no. 512335), and IFN-γ (fluorescein isothiocyanate) (clone 4S.B3, catalog no. 502505) according to the manufacturer’s instructions (BioLegend, San Diego, CA). The cells were analyzed on a FACS analyzer (BD LSRFortessa). Electronic gates were placed on live CD4^+^ or CD8^+^ cells using FlowJo software (BD Biosciences, Ashland, OR), and the proportions of CD25^+^ or IFN-γ^+^ cells were measured. The percentage positive was determined by comparison to an isotype control antibody.

### Statistical analyses

Analyses were performed using GraphPad Prism version 8.2.1 software (GraphPad Software Inc., San Diego, CA) and SAS (version 9.4, SAS Cary NC). All cytokine data were log transformed for analysis. Groups were compared using Fisher exact test or χ^2^ test, ANOVA with Tukey’s post hoc correction, or multiple regression analysis with a mixed model for repeated measure (Sidak’s multiple comparison). A *P* value <0.05 was considered significant. Missing data were due to absence of clinical laboratory data or insufficient serum or peripheral blood samples. Unless indicated, the mean ± SD and raw data are shown.

## References

[1] Johns Hopkins Coronavirus Resource Center, COVID-19 United States Cases by County (Johns Hopkins University, 2020); https://coronavirus.jhu.edu/us-map [accessed 2020 August 11].

[2] E. G. Price-Haywood, J. Burton, D. Fort, L. Seoane, Hospitalization and mortality among black patients and white patients with COVID-19. N. Engl. J. Med. 382, 2534–2543 (2020). 32459916 10.1056/NEJMsa2011686 PMC7269015

[3] S. Richardson, J. S. Hirsch, M. Narasimhan, J. M. Crawford, T. McGinn, K. W. Davidson; Northwell COVID-19 Research Consortium, D. P. Barnaby, L. B. Becker, J. D. Chelico, S. L. Cohen, J. Cookingham, K. Coppa, M. A. Diefenbach, A. J. Dominello, J. Duer-Hefele, L. Falzon, J. Gitlin, N. Hajizadeh, T. G. Harvin, D. A. Hirschwerk, E. J. Kim, Z. M. Kozel, L. M. Marrast, J. N. Mogavero, G. A. Osorio, M. Qiu, T. P. Zanos, Presenting characteristics, comorbidities, and outcomes among 5700 patients hospitalized with COVID-19 in the New York City Area. JAMA 323, 2052–2059 (2020). 32320003 10.1001/jama.2020.6775 PMC7177629

[4] NYC Dept of Health and Mental Hygiene, "*NYC Coronavirus Disease 2019 (COVID-19) Data*" (New York City, 2020).

[5] R. Cagliani, D. Forni, M. Clerici, M. Sironi, Computational inference of selection underlying the evolution of the novel coronavirus, Severe Acute Respiratory Syndrome Coronavirus 2. J. Virol. 94, e00411-20 (2020). 32238584 10.1128/JVI.00411-20 PMC7307108

[6] Coronavirus Disease 2019 in Children – United States, Feb 12-April 2, 2020 (Morbidity and Mortality Weekly Reports, Centers for Disease Control and Prevention, Atlanta, GA, 2020).

[7] W. R. Otto, S. Geoghegan, L. C. Posch, L. M. Bell, S. E. Coffin, J. S. Sammons, R. M. Harris, A. R. Odom John, X. Luan, J. S. Gerber, The epidemiology of severe acute respiratory syndrome coronavirus 2 in a pediatric healthcare network in the united states. J. Pediatric Infect. Dis. Soc., piaa074 (2020). 10.1093/jpids/piaa074 PMC7337783 32559282

[8] Y. Dong, X. Mo, Y. Hu, X. Qi, F. Jiang, Z. Jiang, S. Tong, Epidemiological characteristics of 2143 pediatric patients with 2019 coronavirus disease in china. Pediatrics 10.1542/peds.2020-0702 (2020).

[9] T. Shi, D. McAllister, K. L. O’Brien, E. A. F. Simoes, S. A. Madhi, B. D. Gessner, F. P. Polack, E. Balsells, S. Acacio, C. Aguayo, I. Alassani, A. Ali, M. Antonio, S. Awasthi, J. O. Awori, E. Azziz-Baumgartner, H. C. Baggett, V. L. Baillie, A. Balmaseda, A. Barahona, S. Basnet, Q. Bassat, W. Basualdo, G. Bigogo, L. Bont, R. F. Breiman, W. A. Brooks, S. Broor, N. Bruce, D. Bruden, P. Buchy, S. Campbell, P. Carosone-Link, M. Chadha, J. Chipeta, M. Chou, W. Clara, C. Cohen, E. de Cuellar, D. A. Dang, B. Dash-Yandag, M. Deloria-Knoll, M. Dherani, T. Eap, B. E. Ebruke, M. Echavarria, C. C. de Freitas Lázaro Emediato, R. A. Fasce, D. R. Feikin, L. Feng, A. Gentile, A. Gordon, D. Goswami, S. Goyet, M. Groome, N. Halasa, S. Hirve, N. Homaira, S. R. C. Howie, J. Jara, I. Jroundi, C. B. Kartasasmita, N. Khuri-Bulos, K. L. Kotloff, A. Krishnan, R. Libster, O. Lopez, M. G. Lucero, F. Lucion, S. P. Lupisan, D. N. Marcone, J. McCracken, M. Mejia, J. C. Moisi, J. M. Montgomery, D. P. Moore, C. Moraleda, J. Moyes, P. Munywoki, K. Mutyara, M. P. Nicol, D. J. Nokes, P. Nymadawa, M. T. da Costa Oliveira, H. Oshitani, N. Pandey, G. Paranhos-Baccalà, L. N. Phillips, V. S. Picot, M. Rahman, M. Rakoto-Andrianarivelo, Z. A. Rasmussen, B. A. Rath, A. Robinson, C. Romero, G. Russomando, V. Salimi, P. Sawatwong, N. Scheltema, B. Schweiger, J. A. G. Scott, P. Seidenberg, K. Shen, R. Singleton, V. Sotomayor, T. A. Strand, A. Sutanto, M. Sylla, M. D. Tapia, S. Thamthitiwat, E. D. Thomas, R. Tokarz, C. Turner, M. Venter, S. Waicharoen, J. Wang, W. Watthanaworawit, L. M. Yoshida, H. Yu, H. J. Zar, H. Campbell, H. Nair; RSV Global Epidemiology Network, Global, regional, and national disease burden estimates of acute lower respiratory infections due to respiratory syncytial virus in young children in 2015: A systematic review and modelling study. Lancet 390, 946–958 (2017). 28689664 10.1016/S0140-6736(17)30938-8 PMC5592248

[10] R. M. Viner, E. Whittaker, Kawasaki-like disease: Emerging complication during the COVID-19 pandemic. Lancet 395, 1741–1743 (2020). 32410759 10.1016/S0140-6736(20)31129-6 PMC7220168

[11] L. R. Feldstein, E. B. Rose, S. M. Horwitz, J. P. Collins, M. M. Newhams, M. B. F. Son, J. W. Newburger, L. C. Kleinman, S. M. Heidemann, A. A. Martin, A. R. Singh, S. Li, K. M. Tarquinio, P. Jaggi, M. E. Oster, S. P. Zackai, J. Gillen, A. J. Ratner, R. F. Walsh, J. C. Fitzgerald, M. A. Keenaghan, H. Alharash, S. Doymaz, K. N. Clouser, J. S. Giuliano Jr., A. Gupta, R. M. Parker, A. B. Maddux, V. Havalad, S. Ramsingh, H. Bukulmez, T. T. Bradford, L. S. Smith, M. W. Tenforde, C. L. Carroll, B. J. Riggs, S. J. Gertz, A. Daube, A. Lansell, A. C. Munoz, C. V. Hobbs, K. L. Marohn, N. B. Halasa, M. M. Patel, A. G. Randolph; Overcoming COVID- Investigators; CDC COVID- Response Team, Multisystem inflammatory syndrome in U.S. children and adolescents. N. Engl. J. Med. 383, 334–346 (2020). 32598831 10.1056/NEJMoa2021680 PMC7346765

[12] L. Liu, Q. Wei, Q. Lin, J. Fang, H. Wang, H. Kwok, H. Tang, K. Nishiura, J. Peng, Z. Tan, T. Wu, K. W. Cheung, K. H. Chan, X. Alvarez, C. Qin, A. Lackner, S. Perlman, K. Y. Yuen, Z. Chen, Anti-spike IgG causes severe acute lung injury by skewing macrophage responses during acute SARS-CoV infection. JCI Insight 4, e123158 (2019). 10.1172/jci.insight.123158 PMC6478436 30830861

[13] A. C. Monsalvo, J. P. Batalle, M. F. Lopez, J. C. Krause, J. Klemenc, J. Z. Hernandez, B. Maskin, J. Bugna, C. Rubinstein, L. Aguilar, L. Dalurzo, R. Libster, V. Savy, E. Baumeister, L. Aguilar, G. Cabral, J. Font, L. Solari, K. P. Weller, J. Johnson, M. Echavarria, K. M. Edwards, J. D. Chappell, J. E. Crowe Jr., J. V. Williams, G. A. Melendi, F. P. Polack, Severe pandemic 2009 H1N1 influenza disease due to pathogenic immune complexes. Nat. Med. 17, 195–199 (2011). 21131958 10.1038/nm.2262 PMC3034774

[14] S. M. Sawyer, P. S. Azzopardi, D. Wickremarathne, G. C. Patton, The age of adolescence. Lancet Child Adolesc. Health 2, 223–228 (2018). 30169257 10.1016/S2352-4642(18)30022-1

[15] Multisystem Inflammatory Syndrome in Children (MIS-C) Associated with Coronavirus Disease 2019 (COVID-19) (CDC Health Alert Network, CDCHAN-00432, Centers for Disease Control and Prevention, Atlanta, GA, 2020).

[16] G. Chen, D. Wu, W. Guo, Y. Cao, D. Huang, H. Wang, T. Wang, X. Zhang, H. Chen, H. Yu, X. Zhang, M. Zhang, S. Wu, J. Song, T. Chen, M. Han, S. Li, X. Luo, J. Zhao, Q. Ning, Clinical and immunologic features in severe and moderate Coronavirus Disease 2019. J. Clin. Invest. 130, 2620–2629 (2020). 32217835 10.1172/JCI137244 PMC7190990

[17] A. Grifoni, J. Sidney, Y. Zhang, R. H. Scheuermann, B. Peters, A. Sette, A sequence homology and bioinformatic approach can predict candidate targets for immune responses to SARS-CoV-2. Cell Host Microbe 27, 671–680.e2 (2020). 32183941 10.1016/j.chom.2020.03.002 PMC7142693

[18] A. Grifoni, D. Weiskopf, S. I. Ramirez, J. Mateus, J. M. Dan, C. R. Moderbacher, S. A. Rawlings, A. Sutherland, L. Premkumar, R. S. Jadi, D. Marrama, A. M. de Silva, A. Frazier, A. F. Carlin, J. A. Greenbaum, B. Peters, F. Krammer, D. M. Smith, S. Crotty, A. Sette, Targets of T cell responses to SARS-CoV-2 coronavirus in humans with COVID-19 disease and unexposed individuals. Cell 181, 1489–1501.e15 (2020). 32473127 10.1016/j.cell.2020.05.015 PMC7237901

[19] Y. Gonzalez, M. T. Herrera, E. Juárez, M. A. Salazar-Lezama, K. Bobadilla, M. Torres, CD161 expression defines a Th1/Th17 polyfunctional subset of resident memory T lymphocytes in bronchoalveolar cells. PLOS ONE 10, e0123591 (2015). 25906076 10.1371/journal.pone.0123591 PMC4408072

[20] J. S. Rathore, Y. Wang, Protective role of Th17 cells in pulmonary infection. Vaccine 34, 1504–1514 (2016). 26878294 10.1016/j.vaccine.2016.02.021

[21] M. Sharma, S. Sharma, S. Roy, S. Varma, M. Bose, Pulmonary epithelial cells are a source of interferon-γ in response to *Mycobacterium tuberculosis* infection. Immunol. Cell Biol. 85, 229–237 (2007). 17310225 10.1038/sj.icb.7100037

[22] R. D. Molony, J. T. Nguyen, Y. Kong, R. R. Montgomery, A. C. Shaw, A. Iwasaki, Aging impairs both primary and secondary RIG-I signaling for interferon induction in human monocytes. Sci. Signal. 10, eaan2392 (2017). 29233916 10.1126/scisignal.aan2392 PMC6429941

[23] A. C. Shaw, D. R. Goldstein, R. R. Montgomery, Age-dependent dysregulation of innate immunity. Nat. Rev. Immunol. 13, 875–887 (2013). 24157572 10.1038/nri3547 PMC4096436

[24] A. K. Azkur, M. Akdis, D. Azkur, M. Sokolowska, W. Veen, M. C. Brüggen, L. O’Mahony, Y. Gao, K. Nadeau, C. A. Akdis, Immune response to SARS-CoV-2 and mechanisms of immunopathological changes in COVID-19. Allergy 75, 1564–1581 (2020). 32396996 10.1111/all.14364 PMC7272948

[25] J. S. Lee, S. Park, H. W. Jeong, J. Y. Ahn, S. J. Choi, H. Lee, B. Choi, S. K. Nam, M. Sa, J. S. Kwon, S. J. Jeong, H. K. Lee, S. H. Park, S. H. Park, J. Y. Choi, S. H. Kim, I. Jung, E.-C. Shin, Immunophenotyping of COVID-19 and influenza highlights the role of type I interferons in development of severe COVID-19. Sci. Immunol. 5, eabd1554 (2020). 32651212 10.1126/sciimmunol.abd1554 PMC7402635

[26] T. Zohar, G. Alter, Dissecting antibody-mediated protection against SARS-CoV-2. Nat. Rev. Immunol. 20, 392–394 (2020). 32514035 10.1038/s41577-020-0359-5 PMC7278217

[27] W. Dejnirattisai, P. Supasa, W. Wongwiwat, A. Rouvinski, G. Barba-Spaeth, T. Duangchinda, A. Sakuntabhai, V. M. Cao-Lormeau, P. Malasit, F. A. Rey, J. Mongkolsapaya, G. R. Screaton, Dengue virus sero-cross-reactivity drives antibody-dependent enhancement of infection with zika virus. Nat. Immunol. 17, 1102–1108 (2016). 27339099 10.1038/ni.3515 PMC4994874

[28] M. E. Dieterle, D. Haslwanter, R. H. Bortz III, A. S. Wirchnianski, G. Lasso, O. Vergnolle, S. A. Abbasi, J. M. Fels, E. Laudermilch, C. Florez, A. Mengotto, D. Kimmel, R. J. Malonis, G. Georgiev, J. Quiroz, J. Barnhill, L. A. Pirofski, J. P. Daily, J. M. Dye, J. R. Lai, A. S. Herbert, K. Chandran, R. K. Jangra, A replication-competent vesicular stomatitis virus for studies of SARS-CoV-2 spike-mediated cell entry and its inhibition. Cell Host Microbe 28, 486–496.e6 (2020). 32738193 10.1016/j.chom.2020.06.020 PMC7332447

[29] N. G. Herrera, N. C. Morano, A. Celikgil, G. I. Georgiev, R. J. Malonis, J. H. Lee, K. Tong, O. Vergnolle, A. B. Massimi, L. Y. Yen, A. J. Noble, M. Kopylov, J. B. Bonanno, S. C. Garrett-Thomson, D. B. Hayes, M. Brenowitz, S. J. Garforth, E. T. Eng, J. R. Lai, S. C. Almo, Characterization of the SARS-CoV-2 S protein: Biophysical, biochemical, structural, and antigenic analysis. bioRxiv, 150607 (2020). 10.1021/acsomega.0c03512 PMC7771249 33458462

